# Secreted Frizzled-related protein-1 is a negative regulator of androgen receptor activity in prostate cancer

**DOI:** 10.1038/sj.bjc.6604976

**Published:** 2009-03-10

**Authors:** Y Kawano, S Diez, P Uysal-Onganer, R S Darrington, J Waxman, R M Kypta

**Affiliations:** 1 Prostate Cancer Research Group, Department of Oncology, Imperial College London, DuCane Road, London W12 0NN, UK; 2 Cell Biology and Stem Cells Unit, CIC bioGUNE, Bizkaia 48160, Spain

**Keywords:** sFRP1, prostate cancer, Wnt signal, Frizzled, Wnt5a

## Abstract

Secreted Frizzled-related protein-1 (sFRP1) associates with Wnt proteins and its loss can lead to activation of Wnt/*β*-catenin signalling. It is frequently downregulated in cancer, including prostate cancer, but its function in prostate cancer is unclear because it can increase proliferation of prostate epithelial cells. We investigated the function of sFRP1 in androgen-dependent prostate cancer and found that sFRP1 inhibited androgen receptor (AR) transcriptional activity. In addition, sFRP1 inhibited the proliferation of androgen-dependent LNCaP cells but not of an androgen-independent subline LNCaP-r, suggesting a role in androgen-dependent growth. The inhibition of AR by sFRP1 was unaffected by co-expression of Wnt3a, stabilised *β*-catenin or *β*-catenin shRNA, suggesting it does not involve Wnt/*β*-catenin signalling. Wnt5a also inhibited AR and expression of Wnt5a and sFRP1 together did not further inhibit AR, suggesting that Wnt5a and sFRP1 activate the same signal(s) to inhibit AR. However, sFRP1 inhibition of AR was unaffected by inhibitors of kinases involved in Wnt/Ca^2+^ and Wnt/planar cell polarity non-canonical Wnt signalling. Interestingly, the cysteine-rich domain of sFRP1 interacted with Frizzled receptors expressed in prostate cancer cells, suggesting that sFRP1/Frizzled complexes activate a signal that leads to repression of AR. Taken together, these observations highlight the function of *β*-catenin-independent Wnt signalling in the control of AR activity and provide one explanation for sFRP1 downregulation in prostate cancer.

Wnt signalling is important in a wide variety of biological processes and is frequently misregulated in cancer. Wnt ligands bind to Frizzled and low-density lipoprotein receptor-related protein 5/6 receptors, thereby activating the Wnt/*β*-catenin pathway, also known as the canonical pathway, and the non-canonical planar-cell polarity (PCP) (Boutros *et al*, 1998) and protein kinase C (PKC)/Ca^2+^ pathways (Slusarski *et al*, 1997). In the Wnt/*β*-catenin pathway, stimulation by Wnt ligand leads to *β*-catenin accumulation in the cytoplasm and translocation to the nucleus, where it associates with T-cell receptor/lymphoid enhancer factor-1 (TCF/LEF-1) family transcription factors (Behrens *et al*, 1996; Molenaar *et al*, 1996) and activates target genes such as *c-Myc* (He *et al*, 1998).

Cytoplasmic and/or nuclear *β*-catenin, which is often used as an indicator of activation of the Wnt/*β*-catenin pathway, is observed in up to 71% of advanced prostate tumour specimens (Chesire *et al*, 2002; de la Taille *et al*, 2003; Yardy *et al*, 2008). However, unlike colon cancer, where either inactivating mutations in APC or activating mutations in *β*-catenin are observed in most cases, mutations in intracellular components of the Wnt signalling pathway in prostate cancer are rare. For instance, 5% of prostate tumours harboured activating mutations in *β*-catenin (Voeller *et al*, 1998; Chesire *et al*, 2000), whereas changes in the coding region of Axin1 were recently identified in 6% of advanced prostate cancer (Yardy *et al*, 2008) and inactivating mutations in APC have not been found in prostate cancer patients (Suzuki *et al*, 1994; Watanabe *et al*, 1996).

Wnt signalling is also regulated by Wnt antagonists such as members of the secreted Frizzled-related protein (sFRP) family. Secreted Frizzled-related proteins are glycoproteins that possess two characteristic domains, the cysteine-rich domain (CRD) in the N terminus and the Netrin-like (NTR) domain in the C terminus (Hoang *et al*, 1996; Finch *et al*, 1997; Leyns *et al*, 1997). The CRDs of sFRPs share homology with Frizzled CRDs, and it is thought that the sFRP1 CRD is essential in antagonising Wnt signals by directly binding to Wnts, thereby preventing Wnt interaction with Frizzleds (Lin *et al*, 1997; Bafico *et al*, 1999; Dann *et al*, 2001; Bhat *et al*, 2007). Although the NTR domain of sFRP1 does not associate with Wnts, it is required for maximal Wnt inhibitory activity (Bhat *et al*, 2007). Secreted Frizzled-related proteins are involved in wide-ranging biological phenomena. The *sFRP1* gene is inactivated in many human cancers either as a result of chromosomal deletions (Stoehr *et al*, 2004; Huang *et al*, 2007) or promoter hypermethylation (Suzuki *et al*, 2002; Takada *et al*, 2004; Lodygin *et al*, 2005; Lo *et al*, 2006; Shih *et al*, 2006; Veeck *et al*, 2006; Dahl *et al*, 2007; Huang *et al*, 2007; Nojima *et al*, 2007), and loss of sFRP1 expression contributes to a poor prognosis (Klopocki *et al*, 2004; Veeck *et al*, 2006).

The androgen receptor (AR) is a member of the nuclear receptor superfamily, and is a key regulator of prostate cancer cell proliferation and survival (Dehm and Tindall, 2007). The transcriptional activity of AR is regulated by interaction with various cofactors (reviewed in Chesire and Isaacs, 2003 and Cronauer *et al*, 2003), which include *β*-catenin (Truica *et al*, 2000; Chesire *et al*, 2002; Mulholland *et al*, 2002; Yang *et al*, 2002). Because both AR transcriptional activity and the cytoplasmic and/or nuclear levels of *β*-catenin (Chesire *et al*, 2002; de la Taille *et al*, 2003) are elevated in prostate cancer, crosstalk between AR and Wnt/*β*-catenin signalling pathways may contribute to prostate cancer progression.

We have investigated how the expression level of sFRP family members in prostate cancer cells affects AR signalling. We hypothesised that loss of sFRP leads to activation of canonical Wnt signalling and, as a result, increased AR transcriptional activity. Our results indicate that sFRP1 represses AR, but that the mechanism of repression is independent of Wnt/*β*-catenin signalling.

## Materials and methods

### Cells and reagents

Cell lines were from the American Type Culture Collection (Rockville, MD, USA), except for LNCaP-r cells that were obtained from El-Nasir Lalani (Imperial College London). Cells were grown as previously described (Mazor *et al*, 2004; Zhu *et al*, 2004; Kawano *et al*, 2006). In some experiments normal growth media were replaced with phenol red-free media (Invitrogen, Paisley, UK) containing charcoal-stripped serum (First Link UK, Birmingham, UK) and DHT (Sigma, St Louis, MO, USA). Anti-Myc monoclonal antibody (9E10) was purchased from Sigma. Recombinant sFRP1 was purchased from R&D Systems (Abingdon, UK).

### Plasmids

Expression plasmids for Myc-tagged full-length human sFRP1, sFRP1-ΔCRD and sFRP1-Δ1 were generous gifts from Jeffrey S Rubin (NCI, Bethesda, MD, USA). Expression plasmids for HA-tagged wild-type and S37A mutant form of *β*-catenin were from Stephen Byers (Georgetown University, Washington, DC, USA). 16xSuperTOPFLASH was from Randall Moon (University of Washington, Seattle, WA, USA). For expression plasmids encoding extracellular domains of human Frizzled fused to human IgG1 heavy chain (pSMT2-Fz1-IgG, pSMT2-Fz3-IgG, pSMT2-Fz4-IgG and pSMT2-Fz6-IgG), cDNA was obtained by PCR using human Frizzled cDNAs (Origene Technologies, Rockville, MD, USA). Detailed methods including sequences of primers are available upon request. Other plasmids and reporters used have been described previously (Mazor *et al*, 2004; Zhu *et al*, 2004; Kawano *et al*, 2006).

### Colony formation assays

LNCaP cells (3 × 10^5^ cells per well) or LNCaP-r cells (2 × 10^5^ cells per well) were plated in six-well plates and transfected with 2 *μ*g of expression plasmids encoding sFRP1 derivatives or pcDNA3.1 as a negative control using FuGENE HD (Roche Diagnostics, Burgess Hill, UK). After 2 days (LNCaP), or on the following day (LNCaP-r), all transfected cells (LNCaP) cells or one-third of transfected cells (LNCaP-r) were re-plated in 100 mm tissue culture plates with 500 *μ*g ml^−1^ G418 (Merck Chemicals, Nottingham, UK). After 2–3 weeks, colonies were visualised by crystal violet staining. Colonies more than 2 mm in diameter were counted and the results were plotted on graphs.

### Transcription assays

All cells were transfected in triplicate in 24-well plates unless otherwise stated, and each well of the 24-well plate was transfected with 40 ng pDM-*β*Gal as an internal control, 200 ng firefly luciferase gene driven by various promoter sequences and the expression plasmids as indicated. The total amount of DNA was brought to 400 ng using empty pcDNA3.1 (Invitrogen). To measure AR transcriptional activity, cells were incubated in hormone-depleted medium before transfection, DHT or vehicle was added 24 h after transfection and cells were grown for a further 24 h. Recombinant sFRP1 or kinase inhibitors were added to cells 24 h after transfection of reporters. Transfected cells were incubated with sFRP1 or kinase inhibitors for 25 h in hormone-depleted medium and for an additional 24 h in the presence of DHT. Measurement, normalisation and calculation of luciferase activity were carried out as previously described (Mazor *et al*, 2004).

### Generation of LNCaP/TR-*β*i cells

LNCaP cells expressing the Tet repressor (LNCaP/TR2) (Kawano *et al*, 2006) were transfected with pTER-*β*-catenin (van de Wetering *et al*, 2003) and selected for resistance to both 6 *μ*g ml^−1^ blasticidin and 300 *μ*g ml^−1^ Zeocin™ (Invitrogen). Positive clones were identified by western blotting for *β*-catenin.

### RT–PCR

RT–PCR was performed essentially as described previously (Zhu *et al*, 2004), but with modified PCR parameters (2 min at 94°C, 30 s at 94°C, 30 s at 55°C and 30 s at 72°C for 35 cycles). Primers for human Frizzleds were designed as reported by Sala *et al* (2000) except Frizzled-8 (forward, 5′-AAGACAGGCCAGATCGCTAA-3′; reverse, 5′-GCCATGCCGAAGAAGTAGAC-3′) and GAPDH (forward, 5′-TGTTGCCATCAATGACCCCTT-3′; reverse, 5′-CTCCACGACGTACTCAGCG-3′).

### IP and western analyses

293 cells (2 × 10^5^ cells per well) were plated in six-well plates and transfected with 50 ng sFRP1 derivatives and 950 ng Frizzled-IgG using FuGENE HD. Cells were harvested 48 h following transfection and extracted using lysis buffer (0.5% Triton X-100, 10 mM HEPES (pH 7.4), 150 mM NaCl, 2 mM EDTA, 2 mM EGTA), supplemented with Complete, EDTA-free protease inhibitor cocktail tablets (Roche Diagnostics). Cell extracts were clarified by centrifugation for 15 min at 16 000 **g** at 4°C and processed for protein A/G precipitation on a rotating wheel in a cold room for 1 h. After five washes in lysis buffer, the beads were re-suspended in SDS sample buffer. For western blotting, extracts and IPs were separated by SDS–PAGE, transferred to nitrocellulose membranes and incubated in 5% Fraction V BSA in TBS-T (20 mM Tris (pH 7.5), 100 mM NaCl, 0.1% Tween 20) for 30 min. After probing with antibodies, antigens were visualised using chemiluminescence (ECL; GE Healthcare, Chalfont St Giles, UK).

## Results

### Inhibition of AR transcriptional activity by sFRP1

**Figure 1:**
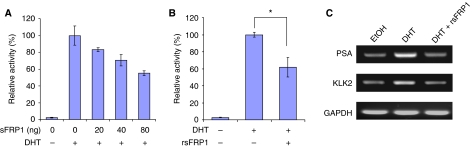
sFRP1 inhibits AR signalling. (**A**) 22Rv1 cells were co-transfected with MMTV-luc, pDM-*β*Gal and increasing amounts of sFRP1 expression plasmid as indicated. At 24 h after transfection, cells were treated with 0.1 nM of the agonist DHT or an equivalent volume of vehicle (ethanol) for 24 h. (**B**) 22Rv1 cells were co-transfected with MMTV-luc and pDM-*β*Gal. At 24 h after transfection, cells were treated with or without 25 μg ml^−1^ recombinant sFRP1 for 5 h, and then further treated with 0.1 nM DHT or an equivalent volume of vehicle (ethanol) for 24 h. Data are average±standard deviation (s.d.) of a representative experiment carried out in triplicate (^*^P<0.005; Student's *t*-test). (**C**) RT–PCR for AR target genes in 22Rv1 cells treated with recombinant sFRP1 and DHT.

**Figure 2:**
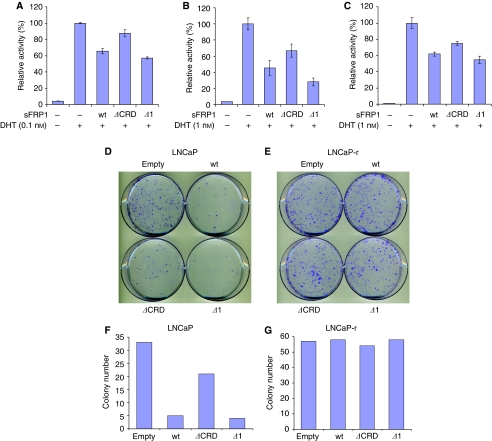
(**A**–**C**) sFRP1 represses AR transcriptional activity and androgen-dependent proliferation of prostate cancer cells principally through the CRD. (**A**) 22Rv1 cells. (**B**) LNCaP cells. (**C**) LNCaP-r cells. All cells were transfected in triplicate in six-well plates, and each well of the six-well plate was transfected with 0.2 *μ*g pDM-*β*Gal as an internal control, 1 *μ*g MMTV-luc and 0.4 *μ*g expression plasmid of sFRP1 derivative. Empty pcDNA3.1 plasmid was used as a negative control. The total amount of DNA was brought to 2 *μ*g using empty pcDNA3.1. At 24 h after transfection, cells were treated with indicated concentration of DHT or an equivalent volume of vehicle (ethanol) for 24 h. Secreted Frizzled-related protein-1 or its derivatives were expressed at comparables level in all cell lines tested (see Supplementary Information 1A–C). (**D**–**G**) sFRP1 reduces colony formation of LNCaP cells but not of the androgen-independent subline LNCaP-r. LNCaP cells (**D** and **F**) or LNCaP-r cells (**E** and **G**) were transfected with sFRP1 or its derivatives, and the number of colonies was determined as described in ‘Experimental procedures’.

We conducted RT–PCR analysis of sFRP family gene expression using cell lines derived from normal prostate and prostate cancer and confirmed previous reports (Lodygin *et al*, 2005) that sFRP1 expression is reduced in prostate cancer cells (data not shown). To study whether sFRP1 could affect AR transcriptional activity, we expressed sFRP1 in 22Rv1 cells, an AR-positive prostate cancer cell line that does not express sFRP family members (data not shown), together with the androgen-responsive reporter MMTV-luciferase and a control reporter, and treated cells with the AR ligand dihydrotestosterone (DHT). As expected, DHT increased AR transcriptional activity (Figure 1A). Interestingly, transfection of sFRP1 expression plasmid repressed AR activity in a dose-dependent manner. To confirm this result in a more physiological context, we repeated these experiments using purified recombinant sFRP1 and found that this also repressed AR transcriptional activity (Figure 1B). In addition, RT–PCR analysis indicated that recombinant sFRP1 reduced expression of the endogenous AR target genes *PSA* and *Kallikrein 2* (*KLK2*) (Figure 1C). These results indicate that sFRP1 has an inhibitory effect on AR transactivation in prostate cancer cells. To investigate which domains of sFRP1 are required for repression of AR, we used sFRP1 mutants lacking either the CRD (ΔCRD) or the C-terminal half containing the NTR domain (Δ1) (Uren *et al*, 2000). Δ1 and wild-type sFRP1 repressed AR to a similar extent, whereas ΔCRD had a significantly weaker effect (Figure 2A). Similar results were observed using LNCaP, another AR-expressing prostate cancer cell line (Figure 2B). These results indicate that the CRD is important for AR repression by sFRP1.

### sFRP1 reduces proliferation of LNCaP cells but not of the androgen-independent subline LNCaP-r

To determine the importance of the CRD in sFRP1 for androgen-dependent prostate cancer cell proliferation, LNCaP cells and a subline of LNCaP, LNCaP-r, which expresses AR but is hormone resistant (Pousette *et al*, 1997), were transfected with empty vector, sFRP1 or the sFRP1 deletion mutants and grown in medium containing G418. Compared to empty vector, full-length sFRP1 and Δ1 significantly inhibited colony formation of LNCaP cells (Figure 2D and F). ΔCRD also reduced colony formation in LNCaP cells, but not to the same extent as full-length sFRP1 and Δ1 (Figure 2D and F). The growth inhibitory effects of the mutants correlated with their effects on AR transcriptional activity in LNCaP cells (Figure 2B). Interestingly, none of the sFRP1 constructs inhibited colony formation of LNCaP-r cells (Figure 2E and G), despite the fact that AR transcriptional activity was similarly inhibited by sFRP1 in LNCaP and LNCaP-r cells (Figure 2B and C). These results indicate that sFRP1 specifically represses colony formation of androgen-dependent prostate cancer cells, and that this repression is mediated principally through the sFRP1 CRD.

### Repression of AR by sFRP1 does not involve Wnt/*β*-catenin signalling

**Figure 3:**
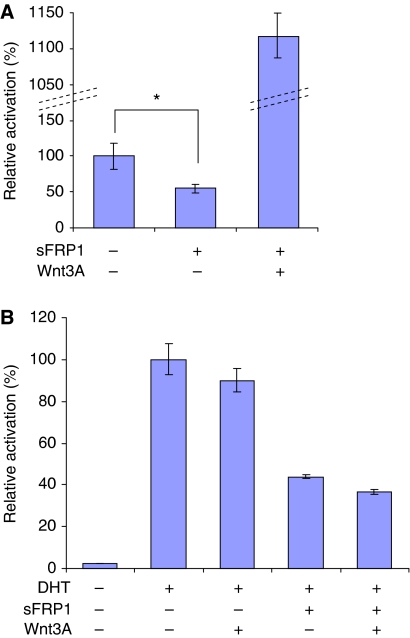
Repression of AR by sFRP1 does not involve Wnt/*β*-catenin signalling. 22Rv1 cells were transfected with luciferase reporter gene (**A**, 16XSuperTOPFLASH; **B**, MMTV-luc), pDM-*β*Gal, 80 ng of sFRP1 plasmid and 80 ng of Wnt3A plasmid as indicated. At 24 h after transfection, cells were treated with 0.1 nM DHT or an equivalent volume of vehicle (ethanol) for 24 h (^*^P<0.00002; Student's *t*-test). Western blotting of cell lysates shows comparable expression of sFRP1 (Supplementary Information 2A). The levels of Wnt3A were too low to be detected by western blotting in these experiments.

Recently, Wnt3a was shown to enhance AR activity at low concentrations of DHT (Verras *et al*, 2004). Because the sFRP1 CRD competes with Frizzled receptors for binding to Wnts (Lin *et al*, 1997; Bafico *et al*, 1999), we hypothesised that sFRP1 represses AR by antagonising autocrine Wnt signals in prostate cancer cells. If this is the case, then excess Wnt3a should rescue the inhibitory effect of sFRP1 on AR. We tested this possibility by conducting luciferase assays using a Wnt-responsive reporter (Figure 3). 22Rv1 cells were found to have low but measurable *β*-catenin/Tcf signalling activity (Figure 3A). Moreover, this activity was repressed by sFRP1 and thus most likely resulted from autocrine signals mediated by endogenous Wnts. The inhibitory effects of sFRP1 on *β*-catenin/Tcf activity were prevented by co-expression of Wnt3a (Figure 3A). We next examined the effect of Wnt3a on sFRP1 repression of AR activity and found that despite preventing sFRP1 repression of *β*-catenin/Tcf activity, Wnt3a did not affect sFRP1 repression of AR activity (Figure 3B). These results suggest that sFRP1 repression of AR is independent of its ability to bind to endogenous Wnt ligands that activate *β*-catenin/Tcf-dependent transcription.

### Repression of AR by sFRP1 does not involve *β*-catenin

**Figure 4:**
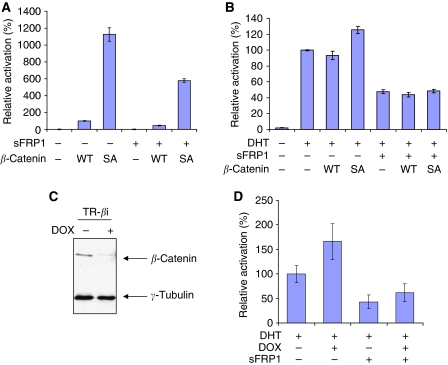
Repression of AR by sFRP1 does not involve *β*-catenin. (**A** and **B**) 22Rv1 cells were transfected with luciferase reporter gene (**A**, 16xSuperTOPFLASH; **B**, MMTV-luc), pDM-*β*Gal, 80 ng of sFRP1 plasmid and 80 ng of wild-type (WT) or S37A mutant (SA) *β*-catenin plasmid as indicated. At 24 h after transfection, cells were treated with 0.1 nM DHT or an equivalent volume of vehicle (ethanol) for 24 h. Western blotting of cell lysates shows comparable expression of sFRP1 and *β*-catenin (Supplementary Information 2B). (**C**) Establishment of an LNCaP subline that expresses a Dox-inducible *β*-catenin shRNA. Doxycycline (Dox, 1 *μ*g ml^−1^) was added to LNCaP/TR-*β*i cells, and cytosolic extracts were analysed for the level of *β*-catenin. *γ*-Tubulin was used as a loading control. (**D**) Following Dox treatment, LNCaP/TR-*β*i cells were transfected with MMTV-luc, pDM-*β*Gal and 80 ng of sFRP1 plasmid. At 24 h after transfection, cells were treated with 0.1 nM DHT or an equivalent volume of vehicle (ethanol) for 24 h.

Secreted Frizzled-related protein-1 can also inhibit *β*-catenin/Tcf activity in colon cancer cells with mutations in APC that stabilise *β*-catenin (Suzuki *et al*, 2004). Moreover, exogenous high-level expression of *β*-catenin increases AR transcriptional activity independently of Tcf/LEF (Truica *et al*, 2000; Chesire *et al*, 2002; Yang *et al*, 2002), suggesting that sFRP1 might repress *β*-catenin/AR activity in a Wnt-independent manner. To investigate this, we compared the effects of sFRP1 on *β*-catenin/Tcf and AR activities in the presence of exogenous *β*-catenin (Figure 4). As expected, expression of wild-type *β*-catenin increased *β*-catenin/Tcf activity, and expression of a stable mutant form of *β*-catenin increased this further (Figure 4A). AR activity was not significantly affected by expression of wild-type *β*-catenin, but it was increased to a small but significant extent by the stable mutant form of *β*-catenin (Figure 4B). Interestingly, sFRP1 reduced the effects of wild-type *β*-catenin and the stable mutant form of *β*-catenin on *β*-catenin/Tcf activity to a similar extent (two-fold). It was clear that both wild-type and the stable mutant forms of *β*-catenin still significantly increased *β*-catenin/Tcf signalling (26-fold and 330-fold higher than endogenous activity respectively) in sFRP1-transfected cells (Figure 4A). Importantly, sFRP1 inhibited AR activity to a similar extent independently of the expression of wild-type and stabilised *β*-catenin (Figure 4B), suggesting that sFRP1 does not act through *β*-catenin to repress AR. To test this further, we established LNCaP cell lines expressing *β*-catenin shRNA in a doxycycline-inducible manner (LNCaP/TR-*β*i cells) (Figure 4C). As we previously reported (Mazor *et al*, 2004), depletion of endogenous *β*-catenin in 22Rv1 cells increased AR activity (Figure 4D), suggesting that the function of endogenous *β*-catenin differs from that of exogenously expressed *β*-catenin. Importantly, depletion of *β*-catenin did not affect sFRP1 repression of AR (Figure 4D), indicating that sFRP1 repression of AR does not require endogenous *β*-catenin. To conclude, the inhibitory effects of sFRP1 on AR do not appear to involve canonical Wnt signalling or *β*-catenin.

### Repression of AR by sFRP1 does not involve kinases implicated in non-canonical Wnt signalling

**Figure 5:**
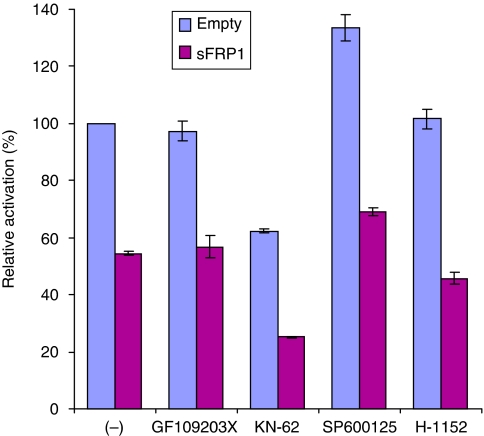
Repression of AR does not involve kinases implicated in non-canonical Wnt signalling. 22Rv1 cells were co-transfected with 80 ng of sFRP1 plasmid, MMTV-luc and pDM-*β*Gal. At 24 h after transfection, cells were treated with GF109203X (2.5 *μ*M), KN-62 (10 *μ*M), SP600125 (10 *μ*M), H-1152 (1 *μ*M) or an equivalent volume of vehicle for 5 h, and then further treated with 0.1 nM DHT for 24 h.

Non-canonical Wnt pathways, such as the PKC/Ca^2+^ and PCP pathways, involve several key kinases, including PKC, calmodulin kinase II (CaMKII), c-Jun N-terminal kinase (JNK) and ROCK, which all have the potential to regulate AR transcriptional activity (de Ruiter *et al*, 1995; Sato *et al*, 1997; Muller *et al*, 2000, 2002; Jeong *et al*, 2004; Backs *et al*, 2006). We reasoned that if sFRP1 repression of AR involves any of these kinases, then inhibitors that target these kinases would not further repress AR in the presence of sFRP1. To test this possibility, 22Rv1 cells were transfected with empty vector or sFRP1 and treated with a panel of kinase inhibitors (Figure 5A). In the absence of sFRP1, the PKC inhibitor GF109203X and the ROCK inhibitor H-1152 did not affect AR transcriptional activity, whereas the CaMKII inhibitor KN-62 repressed AR activity and the JNK inhibitor SP600125 increased AR activity (Figure 5B). Importantly, sFRP1 repressed AR regardless of the presence of any of these kinase inhibitors, with none of the kinase inhibitors significantly reducing the sFRP1 fold repression of AR compared to empty vector. These results suggest that serine/threonine kinases implicated in non-canonical Wnt signalling are not required for sFRP1 repression of AR.

### Interaction of sFRP1 with Frizzled receptors expressed in prostate cancer cells

**Figure 6:**
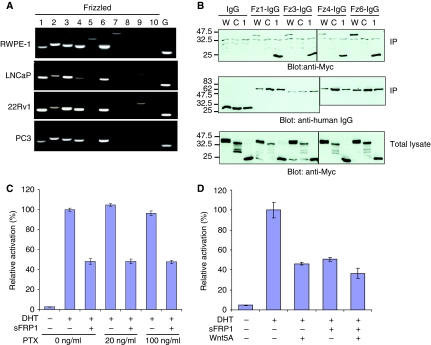
sFRP1 associates with Frizzled receptors expressed in prostate cancer cells. (**A**) RT–PCR analysis of Frizzled expression in normal prostate and prostate cancer cell lines. G, GAPDH. (**B**) 293 cells were co-transfected with sFRP1 derivatives and Frizzled-IgG, then cell lysates were analysed by immunoprecipitation with protein A/G agarose and western blotting with anti-myc directly (top). To confirm immunoprecipitation of Frizzled-IgG, the immunoprecipitated sample was analysed by western blotting with anti-human IgG antibody (middle). To confirm expression of sFRP1 derivatives in cells, total lysate was analysed by western blotting with anti-myc antibody (bottom). W, sFRP1 wild type; C, sFRP1-ΔCRD; 1, sFRP1-Δ1. (**C**) 22Rv1 cells were co-transfected with MMTV-luc and pDM-*β*Gal. At 24 h after transfection, cells were treated with the indicated concentration of PTX or an equivalent volume of vehicle for 5 h, and then further treated with 0.1 nM DHT or an equivalent volume of vehicle (ethanol) for 24 h. (**D**) 22Rv1 cells were co-transfected with 80 ng of sFRP1 plasmid, 80 ng of Wnt5a plasmid, MMTV-luc and pDM-*β*Gal. At 24 h after transfection, cells were treated with 0.1 nM DHT or an equivalent volume of vehicle (ethanol) for 24 h. Western blotting of cell extracts shows comparable expression of sFRP1 and Wnt5a (Supplementary Information 2C).

Recent reports indicate that sFRP1 can also directly interact with Frizzled receptors to trigger intracellular signals (Bafico *et al*, 1999; Rodriguez *et al*, 2005). Therefore, we hypothesised that sFRP1 represses AR by activating a Frizzled-mediated signal. To identify candidate Frizzled receptors that could be involved, RT–PCR was performed using specific primers targeting each Frizzled family member in a panel of normal prostate and prostate cancer cell lines. Frizzled-1, -2, -3, -4 and -6 were expressed in all prostate cell lines examined (Figure 6A). Because Frizzled-2 expression was relatively weak in the AR-expressing prostate cancer cell lines (22Rv1 and LNCaP), Frizzled-1, -3, -4 and -6 were chosen for the further analysis. To explore the possibility that sFRP1 interacts with these Frizzleds, the extracellular domains of these family members (containing the CRD) were fused to the Fc domain of human IgG1 and expressed in HEK 293 cells together with full-length or deletion mutants of sFRP1 (Figure 6B). Immunoprecipitation (IP) analysis showed that all Frizzled–Fc fusion proteins interacted with full-length sFRP1 and Δ1 but not with ΔCRD (Figure 6B). These results indicate that the sFRP1 CRD is required for interaction with Frizzled-1, -3, -4 and -6. Although further studies will be required, these results raise the interesting possibility that sFRP1 repression of AR is mediated by signals acting directly through Frizzleds. Secreted Frizzled-related protein-1 regulates axon growth of retinal ganglion cells through direct binding to Frizzled and activation of a trimeric G-protein pathway that can be blocked by pertussis toxin (PTX) (Rodriguez *et al*, 2005). We therefore tested the effect of repressing trimeric G-protein signals on sFRP1 inhibition of AR by treating 22Rv1 cells with PTX. As shown in Figure 6C, sFRP1 repression of AR was not affected by PTX, suggesting that sFRP1 repression of AR does not involve PTX-sensitive G-protein signals.

These results suggest that the sFRP1/Frizzled interaction might repress AR through a novel signalling pathway. Although we have ruled out canonical and several of the non-canonical Wnt signals in the repression of AR by sFRP1, Wnt5a is thought to activate additional, as yet uncharacterised, signals (Mikels and Nusse, 2006). Therefore, we examined the effect of Wnt5a on AR activity in 22Rv1 cells. Wnt5a repressed AR, and expression of Wnt5a and sFRP1 together did not lead to further inhibition of AR (Figure 6D), suggesting that sFRP1 and Wnt5a activate the same signalling pathway(s) to repress AR.

## Discussion

Secreted Frizzled-related protein-1 expression is downregulated in many cancers including prostate cancer (Ugolini *et al*, 2001; Suzuki *et al*, 2002; Caldwell *et al*, 2004; Klopocki *et al*, 2004; Stoehr *et al*, 2004; Takada *et al*, 2004; Lodygin *et al*, 2005; Lo *et al*, 2006; Shih *et al*, 2006, 2007; Veeck *et al*, 2006; Dahl *et al*, 2007; Huang *et al*, 2007; Nojima *et al*, 2007). Because sFRP1 is a Wnt antagonist and the Wnt/*β*-catenin/TCF axis is aberrantly activated in cancer, it is plausible that downregulation of sFRP1 contributes to abnormal activation of the *β*-catenin/TCF complex. This is indeed the case in some tumours, where restoration of sFRP1 expression inhibits both *β*-catenin/TCF activity and cancer cell growth (Suzuki *et al*, 2004; Nojima *et al*, 2007; Shih *et al*, 2007). However, although both downregulation of sFRP1 and accumulation of cytoplasmic *β*-catenin are frequently observed in prostate cancer (Chesire *et al*, 2002; de la Taille *et al*, 2003; Lodygin *et al*, 2005), *β*-catenin/TCF activity is much lower than in cancers such as colon cancer, in which *β*-catenin/TCF signalling is essential for tumour cell growth (Lodygin *et al*, 2005; YK and RMK, unpublished observations). Therefore, it is important to consider the possibility that loss of sFRP1 affects signalling pathways other than those mediated by *β*-catenin/TCF, and that these drive prostate cancer cell proliferation. In this paper, we have shown that sFRP1 represses AR-dependent transcription both in androgen-dependent LNCaP cells and in the androgen-independent derivative, LNCaP-r. Importantly, sFRP1 inhibited colony formation of LNCaP cells but not of LNCaP-r cells, thus linking the growth inhibitory effects of sFRP1 to androgen-dependent proliferation of prostate cancer cells.

Secreted Frizzled-related protein-1 also reduced the mRNA expression levels of the androgen-regulated genes *PSA* and *KLK2* in 22Rv1 cells. These findings are consistent with a recent report by Joesting *et al* (2005) demonstrating that sFRP1 negatively regulates expression of androgen-regulated proteins by prostate luminal epithelial cells *in vivo*. Joesting *et al* also have shown evidence that proliferation of prostate epithelial cells is reduced in *Sfrp1* null mice and increased in sFRP1 transgenic mice. These observations might appear to contradict our results. However, the function of AR in normal prostate epithelial cells *in vivo* is anti-proliferative (Wu *et al*, 2007), whereas AR has proliferative function in prostate cancer. Thus, the mouse phenotypes may, at least in part, reflect the function of sFRP1 in the regulation of AR transcriptional activity in the normal prostate.

We have found that the sFRP1 mutant comprising the CRD but not the NTR domain (Δ1) inhibited both AR activity and colony formation to the same extent as wild-type sFRP1, whereas the sFRP1 mutant comprising the NTR domain but not the CRD (ΔCRD) had a weaker effect in both assays (Figure 2). These results indicate that the CRD has an important function in the repression of AR by sFRP1. It is intriguing that ΔCRD retains some inhibitory activity. The NTR domain in this mutant has affinity for heparin, and so it is possible that it sequesters heparan-sulphate proteoglycans and inhibits serum growth factors such as FGF-2, which has been shown to regulate androgen-dependent AR activity and LNCaP cell growth (Kassen *et al*, 2000). However, in the context of full-length sFRP1, it is clear that the CRD has the predominant function in repressing AR.

Because sFRP1 is best known as a Wnt antagonist, it is plausible that it represses AR by sequestering endogenous Wnt ligands secreted by prostate cancer cells. Indeed, we have previously reported that prostate cancer cell lines express several Wnt family members (Zhu *et al*, 2004). Therefore, we tested whether Wnt3a, which directly binds to sFRP1 and has been reported to potentiate AR activity in LNCaP cells (Verras *et al*, 2004), could rescue sFRP1 inhibition of AR. In contrast to what was reported for LNCaP cells, Wnt3a did not significantly increase AR activity in 22Rv1 cells (Figure 3B), consistent with a previous report using this cell line (Cronauer *et al*, 2005). Moreover, although co-expression of Wnt3a rescued sFRP1 repression of *β*-catenin/Tcf activity in 22Rv1 cells (Figure 3A), it had no effect on sFRP1 repression of AR activity (Figure 3B). This suggests that repression of AR by sFRP1 does not involve sequestration of endogenous canonical Wnt signals that might be responsible for activating AR.

Similarly, co-expression of *β*-catenin enhanced *β*-catenin/Tcf activity in 22Rv1 cells (Figure 4A), but had no effect on sFRP1 repression of AR activity (Figure 4B). Despite having relatively low *β*-catenin/Tcf activity, prostate cancer cell lines contain significant amounts of cytoplasmic *β*-catenin. Therefore, it was important to determine whether sFRP1 repression of AR required endogenous *β*-catenin. Our experiments using shRNA to deplete *β*-catenin clearly showed that endogenous *β*-catenin is not required for sFRP1 repression of AR (Figure 4C and D).

Secreted Frizzled-related protein-1 can also interact with Wnt proteins that are involved in so-called Wnt non-canonical signalling pathways, including the Ca^2+^/PKC (Slusarski *et al*, 1997) and PCP pathways (Boutros *et al*, 1998). Activation of these pathways is thought to involve PKC, CaMKII (Slusarski *et al*, 1997), JNK (Boutros *et al*, 1998) and ROCK (Marlow *et al*, 2002). Several of these kinases can affect AR signalling: CaMKII phosphorylates and inactivates HDAC4 (Backs *et al*, 2006), which represses AR (Jeong *et al*, 2004), ROCK activates FHL2, a co-activator for AR (Muller *et al*, 2000, 2002) and JNK activates AP-1, which can repress AR (Sato *et al*, 1997). Consistent with some of these reports, both KN-62 (a CaMKII inhibitor) and SP600125 (a JNK inhibitor) affected AR activity in 22Rv1 cells (Figure 3B). However, none of the kinase inhibitors tested was able to mimic or rescue sFRP1 inhibition of AR. Activation of CaMKII and JNK was also monitored in 22Rv1 cells expressing sFRP1 or treated with recombinant sFRP1 by western analysis using phospho-specific antibodies. However, neither kinase was activated by sFRP1 in 22Rv1 cells (YK and RMK, unpublished observations), suggesting that sFRP1 repression of AR is not mediated by the kinases implicated in the Ca^2+^/PKC or PCP pathways.

Recent reports indicate that sFRP proteins can signal independently of Wnts (Bovolenta *et al*, 2008). Rodriguez *et al* (2005), for example, showed that sFRP1-induced axonal outgrowth growth is mediated by a direct interaction between sFRP1 and Frizzled-2. The effects of sFRP1 were mediated by the CRD and involved activation of heterotrimeric G proteins (Rodriguez *et al*, 2005). Although we found that the CRD of sFRP1 is able to interact with each of four frizzled family members that are highly expressed in AR-expressing prostate cancer cell lines, sFRP1 repression of AR was not rescued by PTX treatment, indicating that G proteins are not involved in this phenomenon.

It is intriguing that Wnt5a and sFRP1 inhibited AR to a similar extent. Moreover, co-transfection of sFRP1 and Wnt5a did not produce an additive effect on repression of AR. Wnt5a was recently reported to activate novel signalling pathways (Yamamoto *et al*, 2007; Fukuda *et al*, 2008). There are conflicting data on whether sFRP1 and Wnt5a directly interact (Dennis *et al*, 1999; Wawrzak *et al*, 2007), but it is plausible that sFRP1 and Wnt5a activate a common downstream pathway(s) that leads to AR inhibition. Future work will address whether signals downstream of Frizzleds affect AR function. One possibility is the involvement of Ror1/2 (Hikasa *et al*, 2002; Oishi *et al*, 2003; Fukuda *et al*, 2008), receptor tyrosine kinases that contain a CRD that binds to Wnt5a (Oishi *et al*, 2003) and to Frizzleds (Li *et al*, 2008). The signals downstream of Ror1/2 have not been characterised, but it would be interesting to investigate whether there is a molecular link between Ror1/2 and AR. Alternatively, sFRP1 may inhibit AR through a mechanism that involves the receptor activator of nuclear factor-*κ*B ligand (RANKL). Secreted Frizzled-related protein-1 was reported to inhibit RANKL-dependent osteoclast formation (Hausler *et al*, 2004) and RANKL is found in prostate cancer cells, where it is thought to mediate the effects of prostate tumour cells on osteoclastogenesis *in vivo* (Zhang *et al*, 2001). A more recent report indicates that many cancer cells (including the prostate cancer line LNCaP) express RANK and respond to RANKL (Jones *et al*, 2006).

To summarise, we have shown that sFRP1 represses AR transcriptional activity and, as a result, inhibits proliferation of androgen-dependent prostate cancer cells and that the CRD is mainly responsible for both of these effects. We have addressed the possible mechanisms of action of sFRP1 and demonstrated that repression of AR by sFRP1 does not involve signals mediated by canonical Wnts, *β*-catenin or by kinases implicated in Wnt/Ca^2+^ and Wnt/PCP signalling. Taken together with our demonstration that sFRP1 can associate with Frizzleds expressed in prostate cancer cells, we propose that sFRP1/Frizzled complexes activate a signal that leads to repression of AR and that inactivation of sFRP1 leads to uncontrolled AR activation, which may be a crucial step in prostate cancer progression.
