# Supplementary material for: Secreted Frizzled-related protein-1 is a negative regulator of androgen receptor activity in prostate cancer
Source: Br J Cancer. 2009 Mar 10;100(7):1165–74. doi: 10.1038/sj.bjc.6604976 (PMC2669996; doi:10.1038/sj.bjc.6604976)
Supplement: Supplementary Figures 1 and 2 [file 6604976x1.ppt]

## Slide 1
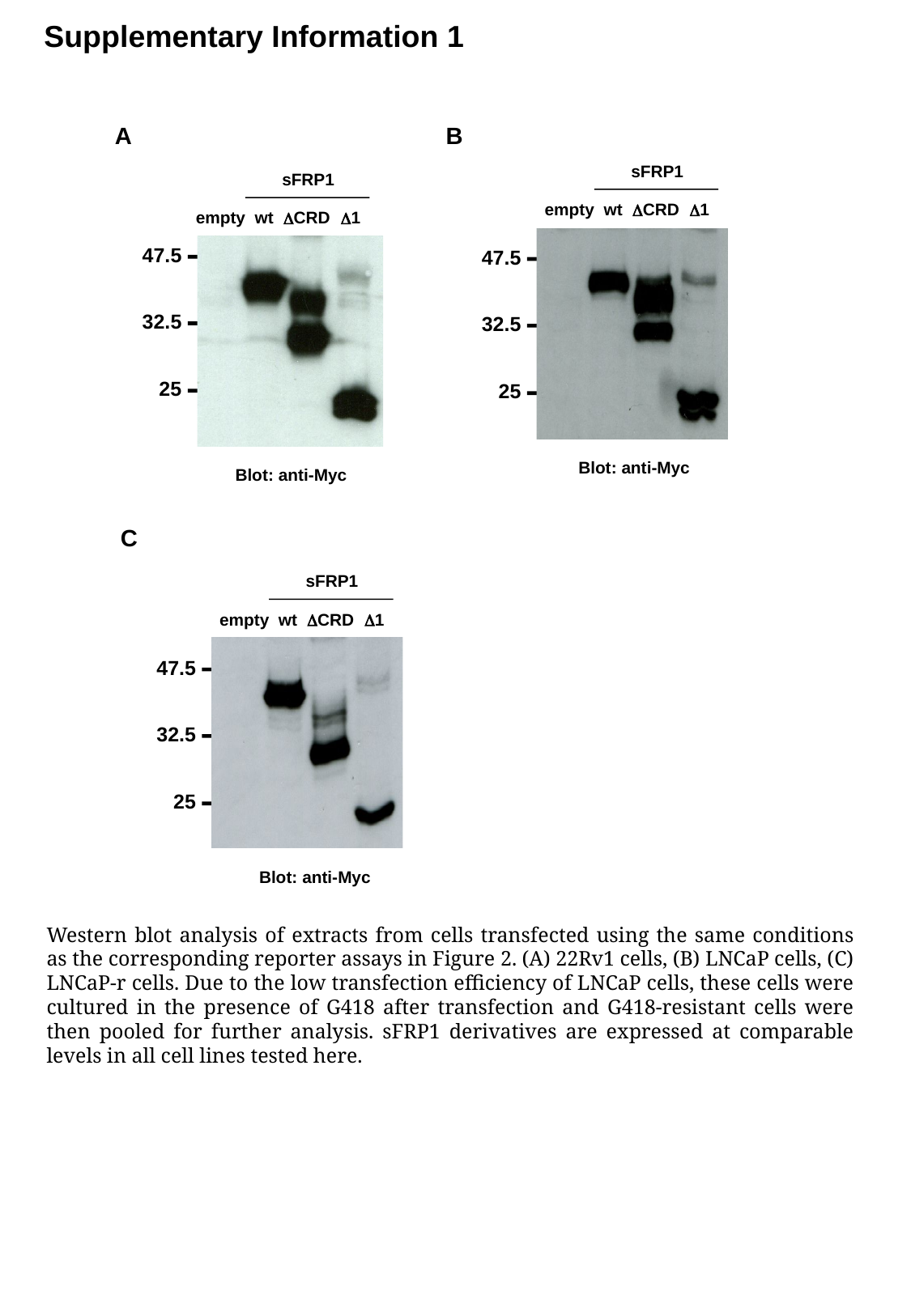

Supplementary Information 1
B
sFRP1
empty
wt
ΔCRD
Δ1
47.5
32.5
25
Blot: anti-Myc
A
sFRP1
empty
wt
ΔCRD
Δ1
47.5
32.5
25
Blot: anti-Myc
C
sFRP1
empty
wt
ΔCRD
Δ1
47.5
32.5
25
Blot: anti-Myc
Western blot analysis of extracts from cells transfected using the same conditions as the corresponding reporter assays in Figure 2. (A) 22Rv1 cells, (B) LNCaP cells, (C) LNCaP-r cells. Due to the low transfection efficiency of LNCaP cells, these cells were cultured in the presence of G418 after transfection and G418-resistant cells were then pooled for further analysis. sFRP1 derivatives are expressed at comparable levels in all cell lines tested here.

## Slide 2
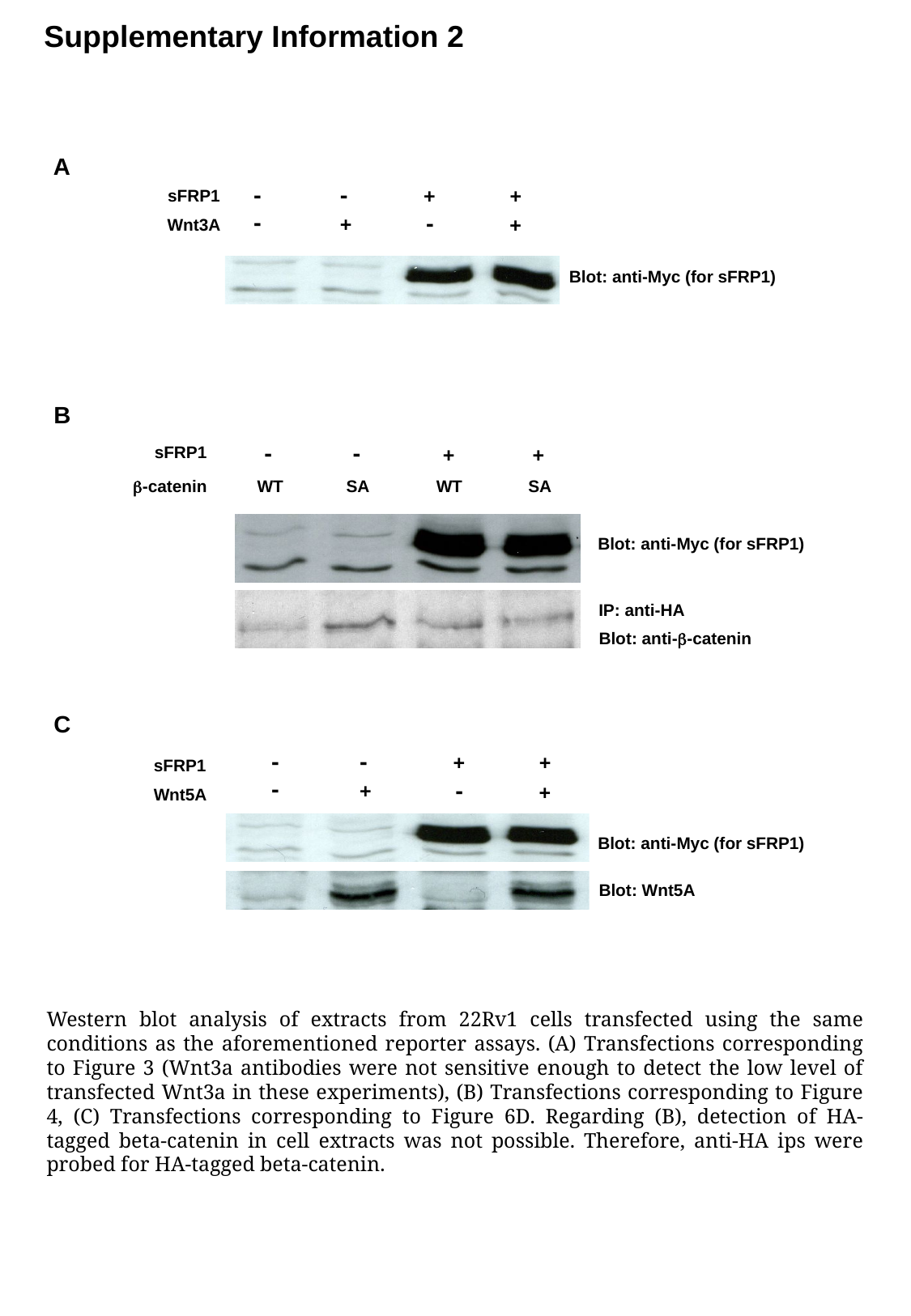

Supplementary Information 2
A
-
-
+
+
sFRP1
-
-
+
+
Wnt3A
Blot: anti-Myc (for sFRP1)
B
-
-
sFRP1
+
+
β-catenin
WT
SA
WT
SA
Blot: anti-Myc (for sFRP1)
IP: anti-HA
Blot: anti-β-catenin
C
-
-
+
+
sFRP1
-
-
+
+
Wnt5A
Blot: anti-Myc (for sFRP1)
Blot: Wnt5A
Western blot analysis of extracts from 22Rv1 cells transfected using the same conditions as the aforementioned reporter assays. (A) Transfections corresponding to Figure 3 (Wnt3a antibodies were not sensitive enough to detect the low level of transfected Wnt3a in these experiments), (B) Transfections corresponding to Figure 4, (C) Transfections corresponding to Figure 6D. Regarding (B), detection of HA-tagged beta-catenin in cell extracts was not possible. Therefore, anti-HA ips were probed for HA-tagged beta-catenin.
